# Selective Interactions of Mouse Melanocortin Receptor Accessory Proteins with Somatostatin Receptors

**DOI:** 10.3390/cells11020267

**Published:** 2022-01-13

**Authors:** Meng Wang, Jing Xu, Xiao-Wei Lei, Cong Zhang, Shang-Yun Liu, Li-Na Jin, Chao Zhang

**Affiliations:** 1 Fundamental Research Center, Shanghai YangZhi Rehabilitation Hospital (Shanghai Sunshine Rehabilitation Center), School of Life Sciences and Technology, Tongji University, Shanghai 201619, China; 1610823@tongji.edu.cn (M.W.); 1911018@tongji.edu.cn (J.X.); 1911002@tongji.edu.cn (X.-W.L.); 2 Department of Plastic and Reconstructive Surgery, Shanghai Institute of Precision Medicine, Shanghai Ninth People’s Hospital, Shanghai Jiao Tong University School of Medicine, Shanghai 200011, China; zczc0627@163.com; 3 Department of Hematology, Changzheng Hospital, Naval Medical University, Shanghai 200041, China; 17717352396@163.com

**Keywords:** somatostatin receptor, G protein-coupled receptors, melanocortin receptor accessory protein 1, heterodimerization

## Abstract

Somatostatin receptors (SSTRs) are G protein-coupled receptors (GPCRs) known to regulate exocrine secretion, neurotransmission, and inhibit endogenous cell proliferation. SSTR subtypes (SSTR1-SSTR5) exhibit homo- or heterodimerization with unique signaling characteristics. Melanocortin receptor accessory protein 1 (MRAP1) functions as an allosteric modulator of melanocortin receptors and some other GPCRs. In this study, we investigated the differential interaction of MRAP1 and SSTRs and examined the pharmacological modulation of MRAP1 on mouse SSTR2/SSTR3 and SSTR2/SSTR5 heterodimerization in vitro. Our results show that the mouse SSTR2 forms heterodimers with SSTR3 and SSTR5 and that MRAP1 selectively interacts with SSTR3 and SSTR5 but not SSTR2. The interactive binding sites of SSTR2/SSTR3 or SSTR2/SSTR5 with MRAP1 locate on SSTR3 and SSTR5 but not SSTR2. The binding sites of MRAP1 to SSTR3 are extensive, while the ones of SSTR5 are restricted on transmembrane region six and seven. The heterodimerization of mouse SSTR2, SSTR3, and SSTR5 can be modulated by binding protein in addition to an agonist. Upregulation of extracellular signal-regulated kinases phosphorylation, p27^Kip1^, and increased cell growth inhibition with the co-expression of SSTR2/SSTR3 or SSTR2/SSTR5 with MRAP1 suggest a regulatory effect of MRAP1 on anti-proliferative response of two SSTR heterodimers. Taken together, these results provide a new insight of MRAP1 on the maintenance and regulation of mouse SSTR dimers which might be helpful to better understand the molecular mechanism involving SSTRs in tumor biology or other human disorders.

## 1. Introduction

As a hypothalamic neuroendocrine hormone, somatostatin exerts its biological functions via five receptor subtypes, somatostatin receptor (SSTR) 1–5. Somatostatinergic system not only plays an important role in different neurological diseases in the central nervous system such as obesity [1] and Alzheimer’s [2], but also modulates endocrine pancreatic functions in the gastrointestinal tract [3,4]. All SSTR subtypes belong to the family of seven transmembrane domain of G protein-coupled receptors (GPCRs) couple to guanine nucleotide-binding protein G(i) subunit alpha [5,6]. GPCRs play a key role in transmitting various extracellular signals into cells and regulating diverse physiological functions. Cyclic adenosine monophosphate (cAMP) inhibition was enhanced upon SSTRs activation [7]. Based on SSTR sequence homology and their pharmacological properties, the receptor subtypes can be classified to two subclasses: somatotropin release inhibiting factor receptor 1 (SRIF1) and somatotropin release inhibiting factor receptor 2 (SRIF2). The SRIF1 receptor group contains SSTR2, SSTR3 and SSTR5, whereas SSTR1 and SSTR4 belong to SRIF2 [8]. SRIF1 and SRIF2 receptors mediate opposite effect on regulating glutamate release [9,10,11]. SRIF1 receptors bind readily to the classical long-acting analogue octreotide, the first analogue used for the treatment of patients with acromegaly or carcinoid tumors [8].

SSTR isoforms display receptor specific homo- and/or heterodimerization within the family or other GPCRs, and the new receptor complexes differ in pharmacological and signaling properties, such as ligand binding, affinity, signal transduction, internalization, and trafficking from the native receptors [12,13,14,15,16,17,18,19,20,21]. Furthermore, the heterodimerization process is highly specific and restricted to SSTR subtypes combinations. For example, human SSTR5 forms heterodimers with SSTR1 and SSTR2 but not with SSTR4 [18]. The clinical implication of SSTRs homo- and heterodimerizations has been shown in several pathophysiological conditions [22,23,24]. However, SSTR subtypes show a great diversity in receptor dimerization in response to agonist. SSTR5 undergoes heterodimerization with SSTR1 upon treatment with agonist [16,18], whereas SSTR2 displays homodimers in the basal condition and dissociates to monomers in the presence of agonist [15]. SSTRs are well known to regulate cell proliferation. The anti-proliferative response of SSTRs involves in the phosphorylation of selective downstream cascades including extracellular signal-regulated kinases (ERK) and the cyclin-dependent kinase inhibitor p27^Kip1^ [25,26,27].

MRAP1 belonging to melanocortin receptor accessory protein (MRAP) family, which is a small protein with conserved amino terminus and a single transmembrane structural domain. In this study, we explored the binding regions for MRAP1 on SSTR3 and SSTR5 proteins. We also characterized MRAP1 as SSTR3 and SSTR5 binding partner negatively affected the dimerization of SSTR2/SSTR3 and SSTR2/SSTR5. More importantly, the dimerization inhibition mediated by MRAP1 seemed to increase the whole activity of SRIF1s when induced by agonist, which was observed through the increasing signaling of cAMP and the shift of ERK1/2 and p27^Kip1^ phosphorylation. In other species, MC4R (Melanocortin Receptor 4) is constitutively active in both cAMP and ERK1/2 pathways, which was differentially regulated by MRAP2a and MRAP2b proteins [28]. Overall, we provide the first evidence that the heterodimerization of mouse SRIF1 receptors can be modulated by MRAP1 protein besides agonist.

## 2. Method and Materials

### 2.1. Plasmids

SSTR1/2/3/5 and MRAP1 were cloned from mouse brain cDNA and inserted in pcDNA3.1(+/−) vectors containing 3HA or 2Flag tag at 5′ terminus. For mutant SSTR with distinct functional domains, desired fragments were cloned from SSTRs and spliced together by overlapping PCR. For fluorescence-activated cell sorting (FACS) assay, SSTRs without TGA were cloned and then inserted into pcDNA3.1(+/−) containing yellow fluorescent protein (YFP)-F1/F2 at 3′ terminus.

### 2.2. Cell Culture and Transfection

Human embryonic kidney (HEK) 293T cells was cultured in Dulbecco’s Modified Eagle’s Medium (DMEM) (HyClone, Logan, UT, USA, SH30243.01) containing 10% fetal bovine serum (FBS) and 1% penicillin/streptomycin at 37 °C, 5% CO_2_ incubator. Transfection was performed when cells grow to cover 70–80% basal area of the dishes and polyethylenimine L (PEI) (Thermo Fisher, Waltham, MA, USA, BMS1003-A) was applied for the transient transfection of desired plasmids.

### 2.3. Western-Blot and Co-Immunoprecipitation (CoIP)

24–48 h upon cell transfection, whole cell protein was extracted from cell culture. Specifically, DMEM was removed and cell lysis (Beyotime, Shanghai, China, P0013) was added to each well. Then, the lysis was transferred to 1.5 mL tubes and rotated for 1 h at 4 °C. One hour later, cells were centrifuged for 15 min at 4 °C 17,000× *g*. Supernatant was transferred to a new tube. 40 µL supernatant was stored as total protein control, and the rest supernatant was immunoprecipitated by anti-HA antibody (Cell Signaling Technology, Inc., Boston, MA, USA, 3724S) overnight at 4 °C. Total protein was stored at −20 °C with protein loading buffer. ProteinA/G beads (Beyotime, P2055) were added to the immunoprecipitation (IP) tubes the next day, and the mixture was rotated for 4 h at 4 °C. Beads were washed three times with cell lysis (containing protease inhibitor) and centrifuged. Finally, loading buffer was added to resuspend the beads. Before SDS-PAGE, total protein and IP supernatant was boiled at 95 °C for 15 min. 12% or 15% gel was used for SDS-PAGE analysis. Anti-HA and anti-Flag antibodies (Cell Signaling Technology, Inc.) were respectively used to blot SSTRs and MRAP1. For ERK and phosphorylated ERK (pERK) test, anti-ERK and anti-pERK (Cell Signaling Technology, Inc.) were respectively used to blot total protein that was directly extracted from cells. For mass spectrum, SDS-PAGE was stained with Coomassie brilliant blue R250 (CBB) (Urchem, Shanghai, China, 20140429) for 1 h at room temperature (RT) subsequently decolorated for 24 h. Then the desired bands were cleaved and sent to Shanghai Omicsspace Biotech Co., Ltd., China for analysis.

### 2.4. Immunofluorescence

Cells were seeded in poly-D-lysine (BBI, Shanghai, China, H412FA0003) treated 12-well plates with slides and transfected with 3HA-GPCR and 2Flag-MRAP1. 24–48 h later, we removed DMEM and added 4% paraformaldehyde (Beyotime, P0099-500 mL) to fix cells. Anti-HA and anti-Flag antibodies (1:1000) were used to incubate cells for 2 h at RT or overnight at 4 °C, then washed three times with Dulbecco’s phosphate-buffered saline (DPBS) (Beyotime, C0021G). Alexa Fluor488 and 647 (Abcam, Cambridge, UK, ab150077 and ab150083) were used as secondary antibodies for HA and Flag respectively. 4′,6-diamidino-2-phenylindole (DAPI) with anti-fade medium (Cell Signaling Technology, Inc., 8961SDAPI) was dropped on glass slides and covered by cell slides. Finally, the slides were observed with Carl Zeiss confocal laser scanning microscope under 63× oil lens. Bimolecular fluorescent complimentary (BiFC) was performed with similar steps without antibody incubation.

### 2.5. FACS Analysis

YFP-F1 and YFP-F2 of YFP fragments were fused with C terminus of SSTR1, SSTR2, SSTR3 and SSTR5, respectively. Cells were seeded in 6-well plates and transfected with SSTR plasmids containing YFP-F1, YFP-F2 and MRAP1 at ratio 1:1:2 the next day. RAMP3 was used as negative control. 24–48 h later, cells were washed once with DPBS after removing DMEM. 1 mL 0.02% EDTA was added to digest cells into single cell at 37 °C, then 1 mL DPBS was added to stop the digestion. Cells were then centrifuged and resuspended in 0.5 mL serum-free DMEM, filtered through a 40 µm mesh sieve into the Polystyrene Round-Bottom Tube (FALCON, Miami, FL, USA). FACS was performed on BD FACSVerse, and FITC channel was used to analyze YFP fluorescent intensity.

### 2.6. Surface Expression Measurement

Cells were seeded in poly-D-lysine treated 24-well plates and transfected with 3HA-GPCR and 2Flag-MRAP1 at different ratios. 24–48 h later, we removed DMEM and added 4% paraformaldehyde to fix cells. After blocking with milk, anti-HA antibody (1:2000) was applied to incubate cells for 2.5 h at RT or overnight at 4 °C, then washed three times with DPBS. Secondary antibody anti-IgG (Cell Signaling Technology, Inc.) was added to each well and held for 2 h at RT, then washed three times with DPBS. Cells were incubated with tetramethylbenzidine (TMB) substrate (Beyotime, P0209-500 mL) for 15–30 min at RT in dark. Finally, the supernatant was transferred to a new 96-well plate to measure luminescence with enzyme-linked immunosorbent assay (ELISA) reader (SpectraMax iD3, Molecular Devices, San Jose, CA, USA). Janus green was used to normalize the cell numbers.

### 2.7. Annexin/Propidium Iodide (PI) Staining

Cells were seeded in 24-well plates and transfected with desired plasmids the next day. 6 h later, the culture media of starvation group was replaced by serum-free DMEM, regular 293T DMEM for other groups. 24 h later, cells were treated with 1 µM octreotide for 30 min at 37 °C. The medium was then discarded and cells were digested with trypsin and washed once with PBS. Then cells were resuspended in 195 µL binding buffer at a density of 2–5 × 10^5^ cells/mL, 5 µL Annexin V-FITC (Sangon Biotech, Shanghai, China, E606336) was added and incubated 10–15 min at RT in dark. Cells were washed in 200 µL binding buffer, and centrifuged at 1000 rpm for 2–5 min to discard supernatant. Finally, cells were resuspended in 190 µL binding buffer, and added 10 µL PI. Analyzing was performed by FACS within 4 h.

### 2.8. MTT Assay

Cell proliferation experiment was performed with 3-[4,5-dimethylthiazol-2-yl]-2,5 diphenyl tetrazolium bromide (MTT) cell proliferation assay kit (Abcam, Ab211091) following the manufacturer’s instructions. Briefly, cell was seeded in 24-well plates coated with poly-D-lysine and transfected with desired plasmids the next day. 6 h later, the culture media of starvation group was replaced by serum-free DMEM, normal 293T DMEM for other groups. 24 h later, cells were treated with 1 µM octreotide for 20 h at 37 °C. Then the medium was discarded and replaced by 200 µL serum-free medium and MTT reagent mixture (1:1). Background control was set with cell-free wells. The whole plates were incubated at 37 °C for 3 h. Then MTT regent was removed, and 300 µL MTT solvent was added into each well. The plates were then shaked for 15 min protected from light. Finally, 200 µL mixture was transferred to a clear 96-well plates and absorbance was measured on ELISA reader at OD = 590 nm.

### 2.9. In Silico and Statistical Analysis

Multi-sequence alignment was conducted by MUSCLE (https://www.ebi.ac.uk/Tools/msa/muscle/ (accessed on 14 November 2021)). The alignments were edited with MView (https://www.ebi.ac.uk/Tools/msa/mview/ (accessed on 20 November 2021)) in which the amino acids were labeled by different colors according to the conservative properties. Results are presented as mean ± S.D of three independent experiments. Statistical analysis was completed by one- or two-way ANOVA and post hoc Dunnett’s or Bonferroni’s tests, as applicable. GraphPad Prism 8.0 (GraphPad Soft- ware, Inc., La Jolla, CA, USA) and FlowJo 10.0 (Becton, Dickinson and Company, Franklin Lakes, NJ, USA) were used for data analysis and *p* value < 0.05 was considered statistically significant. ns (not significant), * *p* < 0.05, ** *p* < 0.01, *** *p* < 0.001, **** *p* < 0.0001. All experiments were repeated at least three times.

## 3. Results

### 3.1. Homology of SSTRs and Selective Interactions with MRAP Proteins

**Figure 1:**
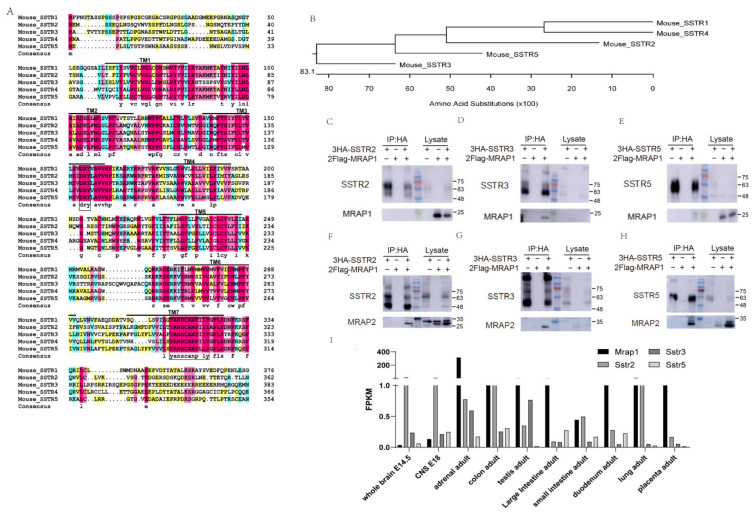
Homology of SSTRs and selective interaction of MRAP1 with SSTRs. (**A**) Multiple sequence alignment of mouse SSTRs (including SSTR1–5), and TM1–7 indicates the seven transmembrane regions of GPCRs. (**B**) Evolutionary distance of SSTR1–5 suggests that SSTR1 is more like to SSTR4, while SSTR2, SSTR3 and SSTR5 are classified into the SRIF1 subgroup. (**C**–**E**) CoIP analysis for the interaction of SRIF1 and MRAP1. Upper membrane is blotted with anti-HA antibody, and the lower is blotted with anti-Flag antibody. The numbers on the right indicate molecular weight of marker band in the middle (kDa). (**F**–**H**) CoIP results for the interaction of SRIF1 and MRAP2. Upper membrane is blotted with anti-HA antibody, and the lower is blotted with anti-Flag antibody. (**I**) Endogenous co-expression of MRAP1 and SSTRs in various tissues. RNA profiling data sets generated by the Mouse ENCODE project.

First, we performed homology analysis of mouse somatostatin receptor family (SSTR1-SSTR5). The five SSTR isoforms displayed the typical molecular architecture shared by GPCRs containing seven transmembrane (TM) regions, the conserved Asp-Arg-Tyr (DRY) motif within TM3 and a highly conserved sequence, YANSCANPI/VLY in TM7, which was recognized as the signature of mammalian somatostatin receptors (Figure 1A). The neighbor joining phylogenetic tree was analyzed based on the amino acid sequences and results showed that in the SRIF2 subgroup, SSTR1 was more similar to SSTR4, while SSTR2, SSTR3 and SSTR5 were classified into the SRIF1 subgroup (Figure 1B).

The MRAP family (MRAP1 and MRAP2) were originally identified to modulate the signaling and dimerization of the melanocortin receptor family in several species [29,30,31,32,33,34]. Recently, they were also found to regulate the trafficking and activity of several other non-melanocortin GPCRs (such as PKR1, OX1R and GHSR1a) [34,35,36,37,38,39]. Here, we transfected HEK293T cells with 3HA-SRIF1s and 2Flag-MRAP1/2 to determine whether MRAP1/MRAP2 could interact with SSTRs. The CoIP assays showed that MRAP1 selectively interacted with SSTR3 and SSTR5 but not SSTR2 (Figure 1C–E), whereas MRAP2 could form complexes with all three members of SRIF1 (SSTR2, 3&5) (Figure 1F–H). GPCRs are usually involved in multiple intracellular signaling pathways, and the binding of agonists exhibit certain signaling bias that has been reported to have potential pathophysiological relevance to various endocrine disorders [40]. Here MRAP1 shows different binding degrees to SSTR2, SSTR3 and SSTR5 suggesting that SSTR3 and SSTR5 may be differentially regulated by MRAP proteins. Moreover, the endogenous co-expression of MRAP1 with SSTR2 and SSTR5 in adrenal, colon and testis indicated the selective interactions of MRAP1 with SSTRs in normal physiological condition (Figure 1I).

### 3.2. Identification of the Binding Regions of MRAP1 with SSTR3 and SSTR5

**Figure 2:**
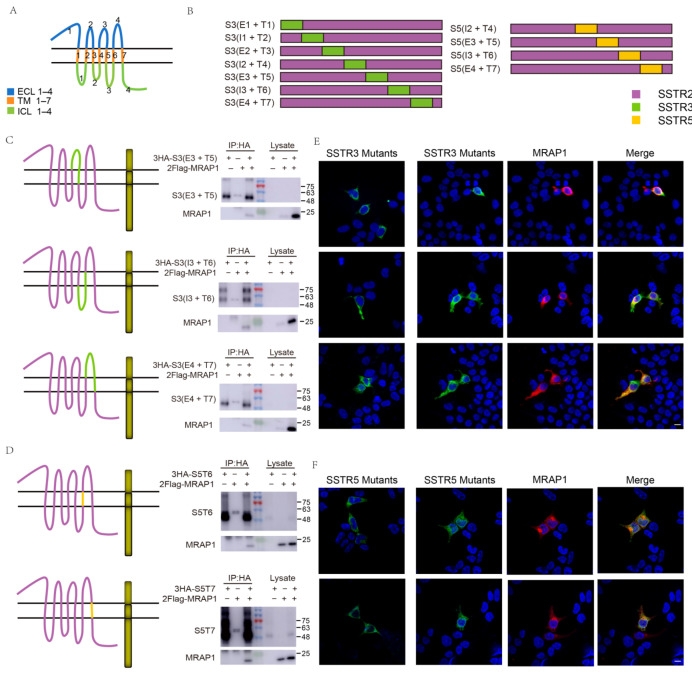
MRAP1 binding regions on SSTR3 and SSTR5. (**A**) Schematic illustration of the domains of GPCR. The regions between adjacent TMs (including the head and tail part) are extracellular loop (ECL, label blue) or intracellular loop (ICLs, label green). Seven transmembrane regions are labeled with orange and called TM. (**B**) Splicing strategy for SSTR mutants. The purple represents region of SSTR2, the green labels region of SSTR3 and the orange labels region of SSTR5. (**C**–**D**) The localization of the functional regions on SSTR3 and SSTR5, which play an important role in interacting with MRAP1. The western blot analysis indicates MRAP1 interacting with following regions: S3(I3 + T6), S3(E3 + T5), S3(E4 + T7), S5T6, S5T7. The numbers on the right indicate molecular weight of marker band in the middle (kDa). (**E**,**F**) Co-immunofluorescence indicates the interaction of SSTR3/5 mutants and MRAP1. The pictures on the left show cells transfected with SSTR3 mutants or SSTR5 mutants, and the pictures on the right show cells co-transfected with SSTR3 mutants (green) and MRAP1 (red) or SSTR5 mutants (green) and MRAP1 (red). Scale bar = 10 µm.

To clarify the selective interactions between MRAP1 and SRIF1s, we then analyzed the binding sites of SSTR3 and SSTR5 for MRAP1. We named the transmembrane regions of SRIF1s as TM1–TM7 (T1–T7), and the regions between two TMs including the extracellular domain and intracellular tail as extracellular loop (ECLs) or intracellular loop (ICLs), respectively (Figure 2A). Due to the fact that MRAP1 showed no interaction with SSTR2, we utilized SSTR2 as a background framework and replaced different regions with the homologous segments of SSTR3 and SSTR5 to screen for the functional interactive domains of SSTR3 and SSTR5.

Firstly, ECL1-TM3 or ICL2-ICL4 of SSTR2 were replaced by the corresponding regions of SSTR3 or SSTR5 to briefly determine the location of binding sites. The results showed that replacement of the two halves of SSTR3 and the C-terminal half of SSTR5 exhibited strong interaction with MRAP1 (Figure S1A,B,J,K). Next, we generated a series of SSTR3 and SSTR5 mutants where the respective TM and its adjacent regions were replaced by the homologous segments of SSTR2 (Figure 2B). Since the N-terminal half of SSTR5 didn’t participate in the interactive binding with MRAP1, we only carried out the replacing strategy for its C-terminal half. Subsequently, we replaced each TM region of SSTR2 separately with the counterpart of SSTR3 or SSTR5 (Figure S1 and Table 1). CoIP and immunofluorescence results suggested that the binding of SSTR3 was restricted to several regions in the C-terminus, while the binding region on SSTR5 was further located in TM6 and TM7 (Figure 2C,D and  Figure S1). Immunofluorescence also verified the co-localization and protein complex formation of SSTR mutants and MRAP1 (Figure 2E,F). In order to compare the interacting function of TMs, ECLs, and ICLs at SSTR3 N-terminus and C-terminus to MRAP1, we carried out western blot densitometry analysis for CoIP. T-test was carried out on densitometry means of N-terminus (E1 + T1, I1 + T2, E2 + T3) and C-terminus (E3 + T5, I3 + T6, E4 + T7). Although some parts on N-terminus of SSTR3 affected its interaction with MRAP1 as well, their affinity with MRAP1 seemed inferior to the ones of C-terminus (Figure S1S,T).

### 3.3. Inhibition of the Formation of SSTR2/SSTR3, SSTR2/SSTR5 Heterodimers by MRAP1

**Figure 3:**
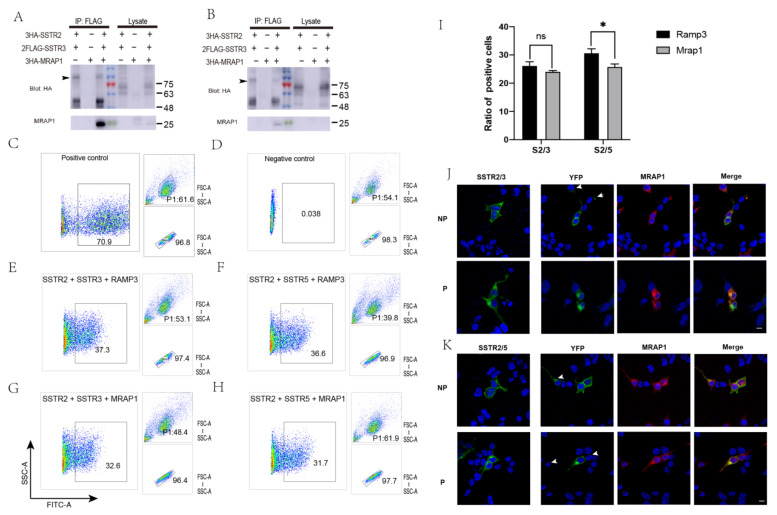
MRAP1 inhibits the formation of SSTR2/3 and SSTR2/5 heterodimers. (**A**,**B**) CoIP of 3HA-SSTR2, 3HA-MRAP1 and 2FLAG-SSTR3 (**A**) or SSTR5 (**B**). The arrow indicates SSTR2/3 and SSTR2/5 heterodimers (~100 kDa). The numbers on the right indicate molecular weight of marker band in the middle (kDa). (**C**–**H**) Bimolecular fluorescence complementation (BiFC) analysis by fluorescence activated cell sorting (FACS). Positive control is transfected with GFP plasmid (**C**) and negative control is transfected with empty pcDNA3.1. MRAP1 significantly decreases the percentage of YFP fluorescent cells in SSTR2/SSTR5 group. (**D**). The number represents the percentage of positive population. (**I**) Statistics for three replicates of FACS. The significance is analyzed by *T*-test. * *p* < 0.05, ns: *p* ≥ 0.05. (**J**–**K**) BiFC for SSTR2 and SSTR3 or SSTR5. The pictures on the left show cells transfected with SSTR2-F1 and SSTR3-F2 or SSTR5-F2, and the ones on the right show cells transfected with MRAP1 besides F1 and F2. The white arrow indicates cells with weak YFP caused by MRAP1. NP: Nonpermeabilized; P: Permeabilized. Scale bar = 10 µm.

Previous reports showed that it was quite common for SSTRs to form functional oligomers [12,41]. And MRAP1 protein exhibited opposite effects on MC2R and MC5R receptor dimerization and trafficking [32]. We speculated that the binding of MRAP1 may also affect the formation of functional dimers SSTR2/SSTR3 and SSTR2/SSTR5. To test this hypothesis, we transfected HEK293T cells with 3HA-SSTR2 and 2Flag-SSTR3 or 2Flag-SSTR5 with or without 3HA-MRAP1 to determine whether MRAP1 could modulate the heterodimerization of SSTR2/SSTR3 and SSTR2/SSTR5. Our results showed that mouse SSTR2 formed heterodimers with SSTR3 and SSTR5 (Figure 3A,B). To further validate the composition of the bands (~100 kDa), we analyzed their components by mass spectrum and the results validated our expectations (Table S1). More importantly, CoIP with differently tagged receptors confirmed that both SSTR2/SSTR3 and SSTR2/SSTR5 dimeric receptor complex co-precipitated with MRAP1 protein. And the presence of MRAP1 posed negative impact on the total amount of these two heterodimers, as indicated by the arrow of Figure 3A,B. This phenomenon was further confirmed by BiFC assay where we co-expressed two receptors that fused to two separate YFP fragments: YFP-F1 or YFP-F1, with or without MRAP1 in HEK293T cells. The YFP fluorescence indicated the presence of SSTR2/SSTR3 or SSTR2/SSTR5 heterodimers on the cell surface. Compared with control groups that co-expressed with RAMP3, another reported transmembrane protein that does not regulate GPCR signaling, counting the fluorescent cells by FACS found that MRAP1 significantly decreased the percentage of YFP fluorescent cells in SSTR2/SSTR5 group (Figure 3C–I). Thus, MRAP1 could inhibit the dimerization of both SSTR2/SSTR3 and SSTR2/SSTR5, and the inhibitive effect was more salient in SSTR2/SSTR5 group.

To further support our FACS results, we co-expressed SSTR2-F1 and SSTR3-F2 or SSTR2-F1 and SSTR5-F2, with or without MRAP1, and performed permeable and non-permeable treatments of the plasma membrane and measured fluorescence with confocal microscope to visualize the effect of MRAP1 on SSTR2/SSTR3 and SSTR2/SSTR5 dimerization in live cells. As expected, in the absence of MRAP1, the SSTR2/SSTR3 dimers exhibited strong fluorescent signal. Some cells showed weak YFP fluorescence when MRAP1 was co-expressed, as indicated by the arrow (Figure 3J). SSTR2/SSTR5 dimers showed the similar results (Figure 3K). We also expected to distinguish differential location of heterodimers in the presence and absence of MRAP1 to illustrate its function in modulating membrane trafficking of SSTR2/SSTR3 and SSTR2/SSTR5 heterodimers. Therefore, we compared permeable groups with and without MRAP1 with the expectation to see more YFP signal locating inside the cells with MRAP1 than the ones without MRAP1. However, no difference was seen between two groups. So, MRAP1 may not regulate this process or its function was too subtle to observe.

### 3.4. Enhancement of MAPK Signaling of SSTR2/3 and SSTR2/5 Heterodimers by MRAP1 Protein

**Figure 4:**
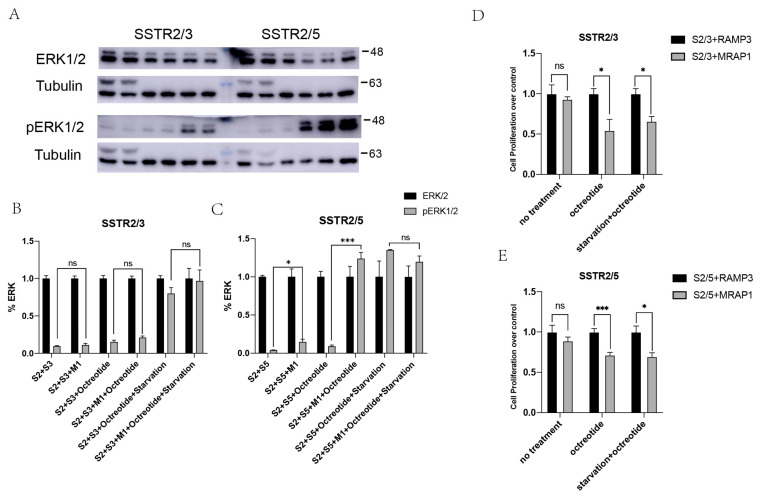
MRAP1 enhances MAPK signaling of SSTR2/3 and SSTR2/5 heterodimers (SSTR2/3, SSTR2/5). (**A**) Western blot for ERK and pERK, and Tubulin is used as reference. The sample order: STRs, SSTRs + MRAP1, SSTRs + octreotide, SSTRs + MRAP1 + octreotide, SSTRs + octreotide + starvation, SSTRs + MRAP1 + octreotide + starvation (from left to right). The numbers on the right indicate molecular weight of marker band in the middle (kDa). The levels of pERK1/2 are significantly increased in the presence of agonist in our double-expressing cells. (**B**,**C**) Quantitative analysis for western blot, *n* = 3. (**D**,**E**) Cell proliferation assay with MTT, *n* = 3. * *p* < 0.05, *** *p* < 0.001, ns: *p* ≥ 0.05.

Phosphorylation of ERK1/2 was shown as a presupposition to the growth-inhibitory effects of somatostatin [42,43]. Moreover, it was shown that the level of pERK1/2 were mediated by SSTR isoforms in a receptor specific manner. To determine the effects of MRAP1 on downstream signaling pathways of SSTR heterodimers, we monitored ERK1/2 phosphorylation upon activation of heterodimers by the treatment of an agonist. As expected, we could see the significantly increased levels of pERK1/2 in the presence of agonist in our dual-expressing cell lines, thus proved the agonist dependent modulation of pERK1/2 cascades. Treatment of HEK293T cells expressing SSTR2, SSTR3 or SSTR5 and MRAP1 with octreotide, a synthetic long-acting cyclic octapeptide with pharmacologic properties similar to somatostatin resulted in a significant increase of phosphorylation compared to control group (Figure 4A–C). We also examined the ERK1/2 phosphorylation of MRAP1 on the SSTR heterodimers with or without FBS. As depicted in Figure 4A, in the absence of FBS, pERK1/2 significantly increased by agonist in comparison to control. In FBS-deficient condition, MRAP1 also significantly enhanced pERK1/2, suggesting that MRAP1 was required to enhance the pharmacological response. Also, this enhancing effect of MRAP1 was more significant in SSTR2/5 group, which was consistent with our BiFC and FACS results in Figure 3. These findings supported the importance of MRAP1 in modulating the intracellular downstream cascades upon activation of a monomer or dimeric SSTRs.

Since ERK pathway was reported to affect cell growth and proliferation, we next assessed the physiological response of SSTR2/SSTR3 and SSTR2/SSTR5 activation on cell proliferation. To determine whether the up-regulation of pERK was associated with changes in cellular physiology, we monitored the cell growth of SSTR heterodimerization co-expressing MRAP1. HEK293T cells co-transfected with SSTR2 and SSTR3 or SSTR2 and SSTR5 were starved for 24 h or incubated with media exposing to agonist for 30 min or non-agonist exposure, then processed for MTT assay. MRAP1 exhibited salient inhibition of cell proliferation than RAMP3 group in cells expressing SSTR2/SSTR3 and SSTR2/SSTR5 respectively (Figure 4D,E), suggesting that the presence of MRAP1 could enhance downstream signaling of SSTR2/SSTR3, SSTR2/SSTR5 heterodimers.

### 3.5. The Role of SSTR2/3 and SSTR2/5 Heterodimers in Cell Apoptosis

Direct effect on cell proliferation of somatostatin through its five different receptor subtypes is conceptually well demonstrated. Recently, the functional consequences of SSTR heterodimers has exhibited anti-proliferative as well [12]. Cyclin dependent kinase p27^Kip1^ leads to cell cycle arrest and is associated with the physiological response of cells towards proliferation in response to somatostatin [12]. In this study, we also wanted to examine the effect of SSTR2/SSTR3 and SSTR2/SSTR5 heterodimers on the levels of cyclin dependent kinase p27^Kip1^ as an index of cell apoptosis. We performed Annexin/PI co-staining to test cell apoptosis, and western blot was performed to check the expression of apoptosis-related protein P27 and P53 (Figure S2). The results suggested that the activation of SRIF1 didn’t affect cell apoptosis.

## 4. Discussion

Some GPCRs possess the ability to form homo- or heterodimers to alter the normal function of these receptors and mediate their response to various physiological stimuli in vivo [44]. The SSTR family mediates diverse functions mainly since one cell or tissue type expresses more than one receptor isoform (as well as other hormonal influences on the complex somatostatin system). Alignment of the amino acid sequences of SSTR1–5 demonstrated that all SSTRs consisted of seven TM domains connected by alternating ECLs and ICLs with an extracellular N-terminus and an intracellular C-terminus (in the same way as other GPCRs). Furthermore, SSTRs all shared some conserved features in TM regions with a DRY motif at the cytoplasmic face of TM3, and a highly conserved sequence, YANSCANPI/VLY in TM7, suggesting that TM regions may be important for binding to receptor-related proteins and other functions. It is considered to have a functional association between SSTR2/SSTR5 heterodimers and rarely tachyphylaxis of patients on somatostatin analogs despite years of continuous administration [20]. SSTR2/SSTR3 mediated cell proliferation and apoptosis has also been proved to understand the molecular interactions involving SSTRs in tumor biology [41]. SSTR2, SSTR3 and SSTR5 all belong to SRIF1 subtypes. SSTR2 and SSTR5 as primary SSTRs expressed in growth hormone secreting pituitary adenomas, are primary targets in somatostatin analogue therapy. Besides, co-expression of SSTR2 and SSTR3 modulates anti-proliferative signaling and apoptosis, which is also benefit to tumor biology research. Thus, the physiological role of SRIF1 dimerization need to be elucidated in detail in the future studies.

So far, agonists are considered to be the principal key factors regulating the formation of SSTR dimers, whereas the other factors affecting dimerization are currently unknown. Here, based on the co-expression of MRAP1 and SRIF in certain tissues and MRAP1 involved in certain characteristics of GPCR dimerization. Interference of MRAP1 may play a role in regulating SRIF dimerization as well. We found that mouse SSTR2 could form heterodimers with SSTR3 and SSTR5, respectively, just like the situation in human studies. In addition, there is a direct interaction between MRAP1 and SSTR2/SSTR3 or SSTR2/SSTR5 heterodimer, which can form a protein complex together in vitro. Moreover, SSTR2/SSTR3 and SSTR2/SSTR5 also form dimers in living cells. The number of fluorescent cells decreased upon the addition of MRAP1, and the weakened YFP fluorescence in the presence of MRAP1 could also be observed under the microscope, which indicated that MRAP1 could inhibit the dimerization of SSTR2/3 and SSTR2/5.

The growth-inhibitory effects of SSTRs have been described in both normal and tumor cells, which has been shown to occur both in vitro and in vivo. SSTR2 has been reported to mediate ERK1/2 activation and the inhibition of cell proliferation [34]. Interestingly, we have found the phosphorylation of ERK and the inhibition of cell proliferation enhanced in the presence of MRAP1. In combination with the obstructing effect of MRAP1 on SRIF1 dimerization, we hypothesized that the dissociation of SRIF1 heterodimers will enhance their total activity in comparison with heterodimers.

In conclusion, our results not only provide the description of SSTR subtypes, SSTR2, SSTR3 and SSTR5, and their roles in the downstream signaling pathways as well as functional consequences of heterodimerizations, but also show the first detailed regulation of intracellular signaling and antiproliferative functions by MRAP1 at least partially attributed to receptor heterodimerization. Our findings might help to better understand the complexities of SSTR receptor family and lead to further identification of a novel therapeutic target in tumor cells expressing SRIF receptors.

## Figures and Tables

**Table 1:** Summary for splicing strategy and CoIP results in Figure S1.

	E1	T1	I1	T2	E2	T3	I2	T4	E3	T5	I3	T6	E4	T7	I4
SSTR1	+														
	+						+								
	−		+		+		+		+		+		+		
SSTR3	+														
	+						+								
	+ (weak)		+		+ (weak)		−		+		+		+		
SSTR5	+														
	−						+								
							−		−		+		+		
							−					+		+	

+: positive interaction with MRAP1; + (weak): weak interaction with MRAP1; −: no interaction with MRAP1.

## Data Availability

Data is contained within the article or supplementary material.

## Supplementary Materials

The following supporting information can be downloaded at: https://www.mdpi.com/article/10.3390/cells11020267/s1. Figure S1: FACS analysis of BiFC to monitor the influence of MRAP1 on SRIF1 dimerization. (A–R) The interaction of MRAP1 and SSTR mutants with distinct functional domains. Refer to the naming as shown in Figure 2. (S–T) Western blot densitometry of SSTR3 CoIP (C–E, G–I), and MRAP1 band was analyzed. T-test was carried out for means of N terminus (E1 + T1, I1 + T2, E2 + T3) and C terminus (E3 + T5, I3 + T6, E4 + T7) in panel S. Two replicates were performed. Figure S2: The functional analysis of SRIF1 heterodimers in apoptosis. (A) Annexin/PI co-staining to test cell apoptosis. The numbers represent the percentage of cell populations. (B) Western-blot for apoptosis-related protein P27 and P53, and GAPDH was used as reference control. SSTRs, SSTRs + MRAP1, SSTRs + octreotide, SSTRs + MRAP1 + octreotide, SSTRs + octreotide + starvation, SSTRs + MRAP1 + octreotide + starvation (from left to right). Table S1: The mass spectrum analysis and the components of the bands indicated by black arrow in Figure 3A,B.
