# Supplementary material for: Selective Interactions of Mouse Melanocortin Receptor Accessory Proteins with Somatostatin Receptors
Source: Cells. 2022 Jan 13;11(2):267. doi: 10.3390/cells11020267 (PMC8773839; doi:10.3390/cells11020267)
Supplement: Supplementary file 1 [file cells-11-00267-s001.zip › cells-1481971-supplementary.pdf]

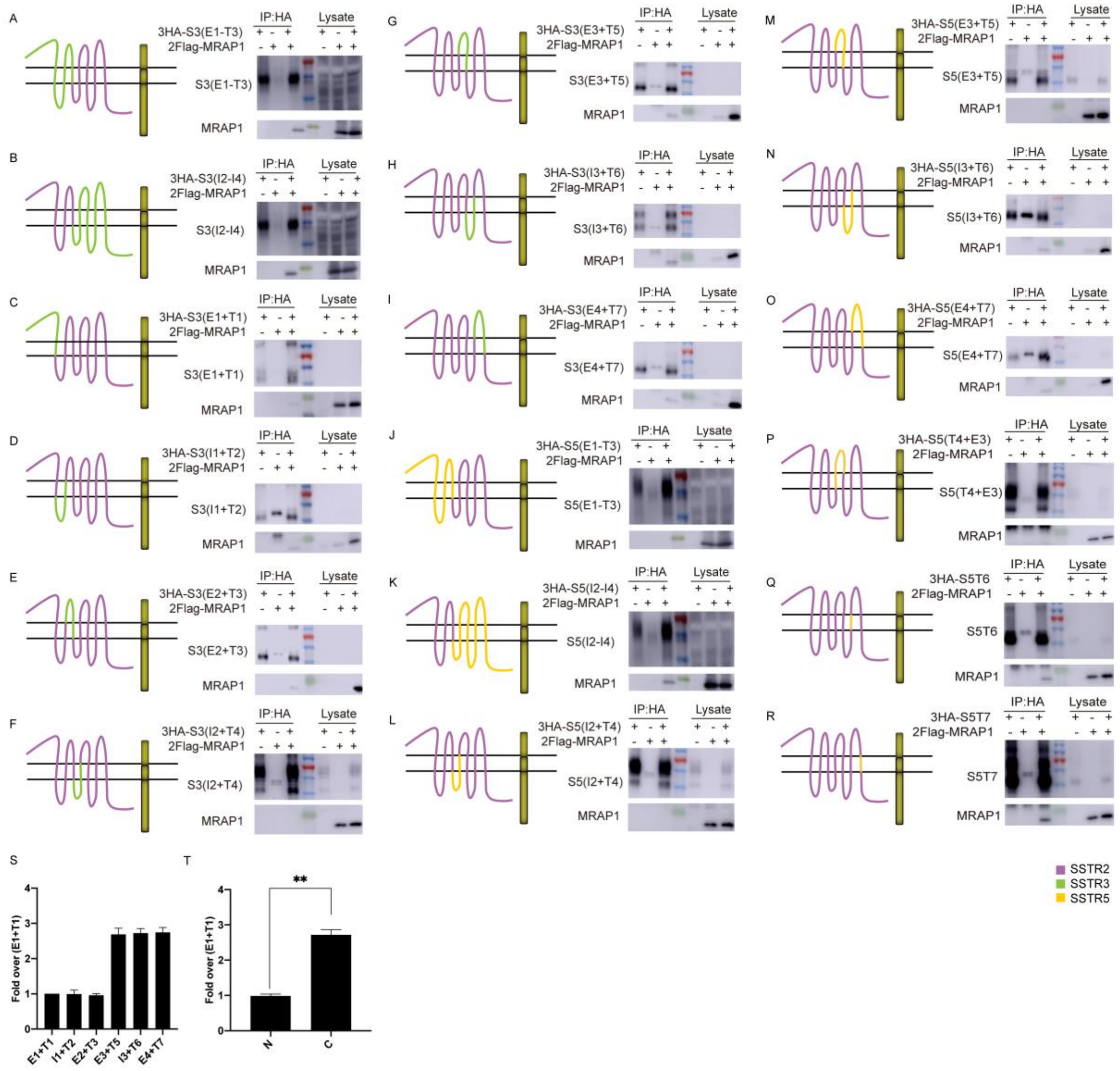

Figure S1—FACS analysis of BiFC to monitor the influence of MRAP1 on SRIF1 dimerization. (A–R) The interaction of MRAP1 and SSTR mutants with distinct functional domains. Refer to the naming as shown in Figure 2. (S–T) Western blot densitometry of SSTR3 CoIP (C-E, G-I), and MRAP1 band was analyzed. T-test was carried out for means of N terminus (E1 + T1, I1 + T2, E2 + T3) and C terminus (E3 + T5, I3 + T6, E4 + T7) in panel S. Two replicates were performed.

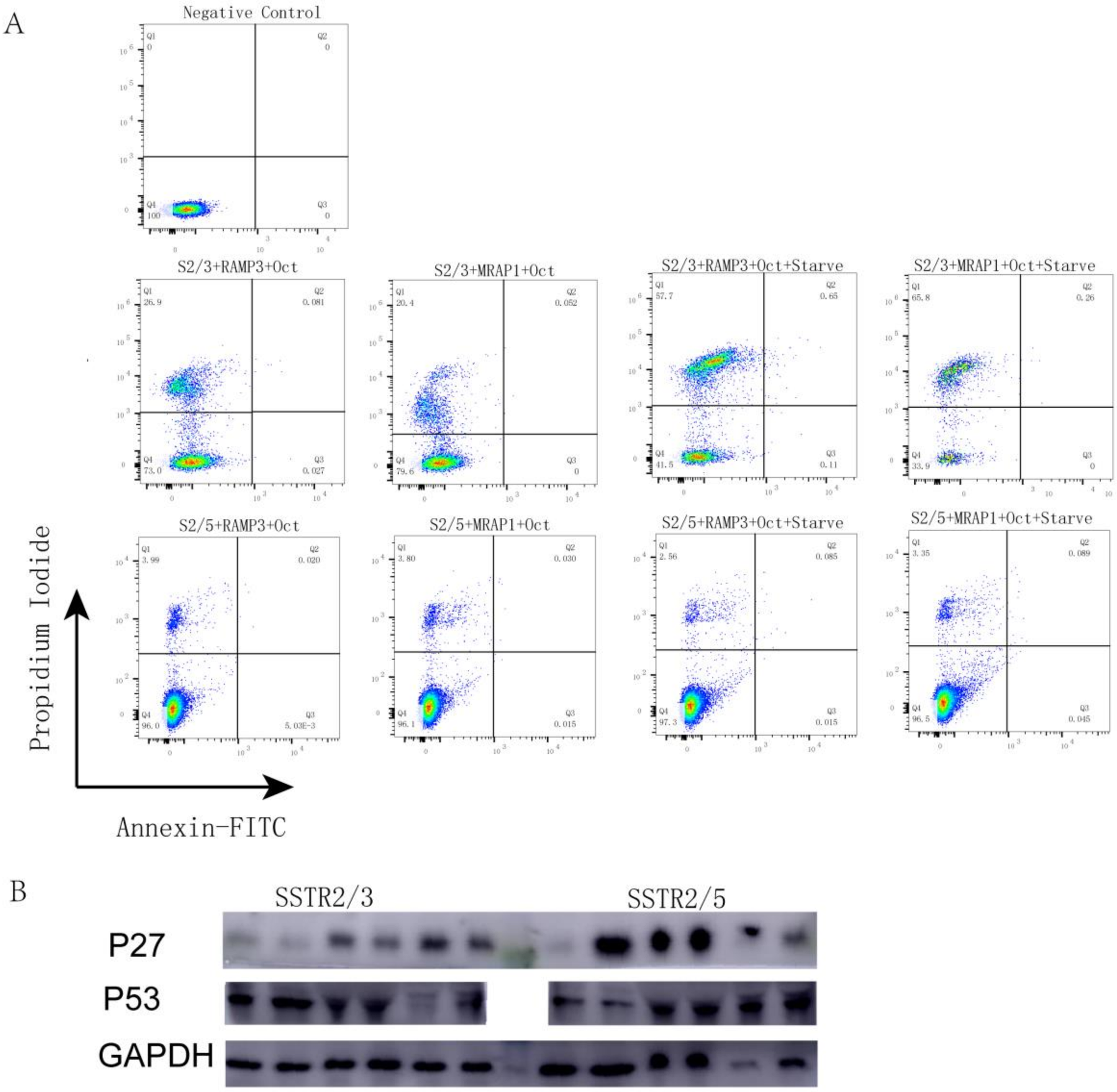

Figure S2—The functional analysis of SRIF1 heterodimers in apoptosis. (A) Annexin/PI co-staining to test cell apoptosis. The numbers represent the percentage of cell populations. (B) Western-blot for apoptosis-related protein P27 and P53, and GAPDH was used as reference control. SSTRs, SSTRs + MRAP1, SSTRs + octreotide, SSTRs + MRAP1 + octreotide, SSTRs + octreotide + starvation, SSTRs + MRAP1 + octreotide + starvation (from left to right).
